# A Novel Endoscopic Approach for Distally Calcified Ureteral Stents

**DOI:** 10.7759/cureus.29427

**Published:** 2022-09-21

**Authors:** Vanessa Aponte, Thomas L Haupt, Rasheed Thompson, Jeremy B Tonkin, Pamela W Coleman

**Affiliations:** 1 Department of Urology, Howard University College of Medicine, Washington, D.C., USA; 2 Department of Urology, Howard University Hospital, Washington, D.C., USA

**Keywords:** urology, bladder calculi, lithotripsy, ureteral stent, endourology

## Abstract

Double-J ureteral stents are an invaluable tool in urology and are one of the most widely used stents in the world. However, when left in situ for prolonged periods, so-called “retained” ureteral stents can lead to numerous complications such as migration, hematuria, encrustation, or stent occlusion. These complications present severe challenges in urologic management. Notably, encrustation of ureteral stents may increase the risk of renal impairment and other potentially life-threatening complications. Here, we present the case of a 34-year-old female with a left double-J ureteral stent who presented to the Emergency Department (ED) with a one-day history of left flank pain and febrile urinary tract infection.

## Introduction

Double-J ureteral stents are one of the most widely used stents in the world. Unfortunately, when left in situ for prolonged periods, they can lead to complications such as encrustation, migration, hematuria, and stent occlusion [1]. Stent encrustation occurs when mineral crystals deposit onto the surface and lumen [2]. Studies have shown that conditions that increase the urinary bacterial load, including recurrent urinary tract infections, diabetes mellitus, and chronic renal failure, can increase the risk of encrustation [3]. Of note, the duration a stent is left in place remains a critical risk factor for encrustation. Endourologic surgery has become the first choice in managing encrusted, retained ureteral stents [4].

Nonetheless, retrieving severely encrusted ureteral stents can be very challenging to manage, and simple cystoscopy and stent extraction may not be possible. This additional complexity may require multiple surgical interventions, thus increasing the risk of iatrogenic complications. Due to this complexity, there is a need to integrate novel multimodal procedures to minimize laser and surgical times and potentially decrease the risk of complications during stent removal, such as stent fracture or ureteral trauma [2]. This case demonstrates an endoscopic, one-session approach to a retained, distally calcified ureteral stent in a 34-year-old female with left flank pain and a febrile urinary tract infection utilizing Olympus ShockPulse-SE (Stone Eliminator) (Olympus Surgical Technologies America, Westborough, Massachusetts, United States) combined with Soltive™ SuperPulsed Laser System (Olympus Surgical Technologies America, Westborough, Massachusetts, United States) lithotripsy.

## Case presentation

Patient and methods

**Figure 1:**
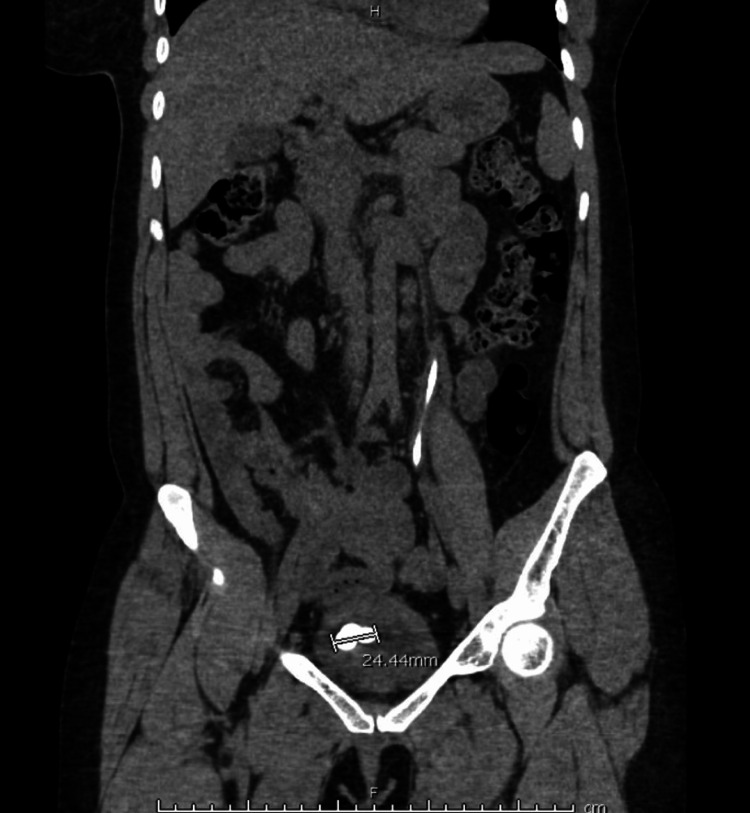
Pre-operative CT abdomen/pelvis further revealed the distally calcified ureteral stent in the urinary bladder

**Figure 2:**
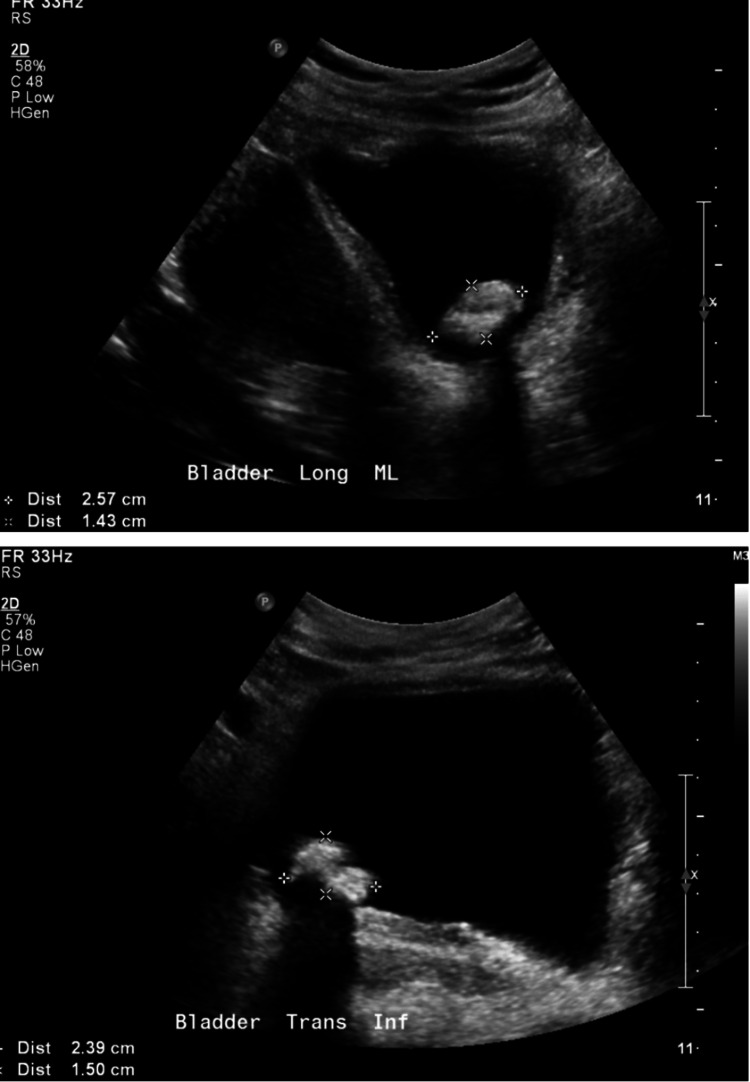
Pre-operative ultrasound further revealed the distally calcified ureteral stent in the urinary bladder

This is a 34-year-old female who presented to the Emergency Department (ED) with a one-day history of left flank pain and febrile urinary tract infection per home measurement. Per the patient, she had placement of a left double-J ureteral stent two years prior, for which she did not follow up for removal due to the COVID-19 pandemic. CT of the abdomen and pelvis without contrast and renal and bladder ultrasound (U/S) was ordered for further evaluation. CT scan (Figure 1) of the abdomen revealed a heavily calcified distal coil of a left double-J ureteral stent with a stone density of 1,457.00 Hounsfield units measuring 24.44 mm and minimal right nephrolithiasis. Renal and bladder U/S (Figure 2) revealed an echogenic calculus within the urinary bladder, causing posterior shadowing measuring 2.6 x 1.4 cm and a second stone measuring 2.4 x 1.5 cm. After obtaining informed consent and following proper preoperative protocol, the patient underwent a combined, one-session approach with cystoscopy and lithotripsy utilizing a combination of Olympus ShockPulse and Soltive laser lithotripsy. In this article, we present a novel, multi-modal, endoscopic approach for the removal of a distally calcified double-J ureteral stent.

In the ED, the patient had a temperature of 98°F, a heart rate of 73 beats/minute, and blood pressure of 109/59 mmHg. Despite the patient being afebrile, her persistent left flank pain was highly concerning for possible pyelonephritis. On urinalysis, there was hematuria with positive nitrites and leukocytes. A complete blood count (CBC), urine culture, blood culture, and basic metabolic panel (BMP) were ordered upon admission. On CBC, there was a mild elevation in the absolute neutrophil count of 7.22 (reference range: (1.3 - 7.1) x10E9). Notably, on BMP, her creatinine level was 1.58. Urine culture revealed 50,000 CFU/mL mixed bacteria flora contamination, and blood cultures resulted in no growth. The patient was promptly started on IV Cefepime 2g q8H and IV fluids for infection control. Over the next few days, the patient’s vital signs remained stable, and her symptoms gradually improved before proceeding with surgical intervention. Due to the feasibility of the procedure at our institution and the fact that it is less invasive than other alternatives, the patient underwent a combined, one-session approach with cystoscopy and lithotripsy utilizing a combination of Olympus ShockPulse and Soltive laser lithotripsy. We found the ShockPulse to be the most effective for the larger stones. The smaller stones were instead eradicated with the Soltive laser.

Procedure and results

**Figure 3:**
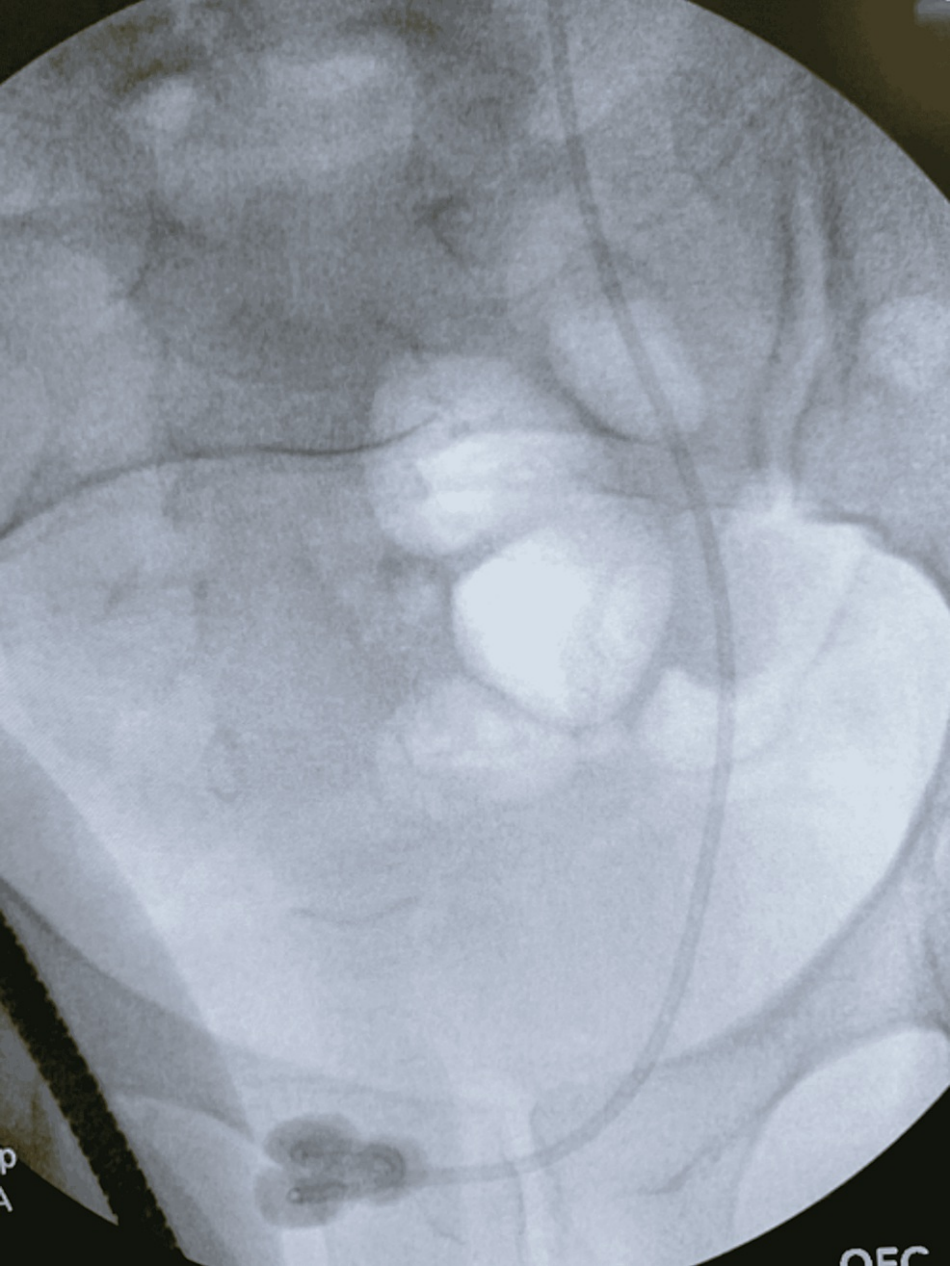
Intraoperative fluoroscopic image of the distally calcified stent

**Figure 4:**
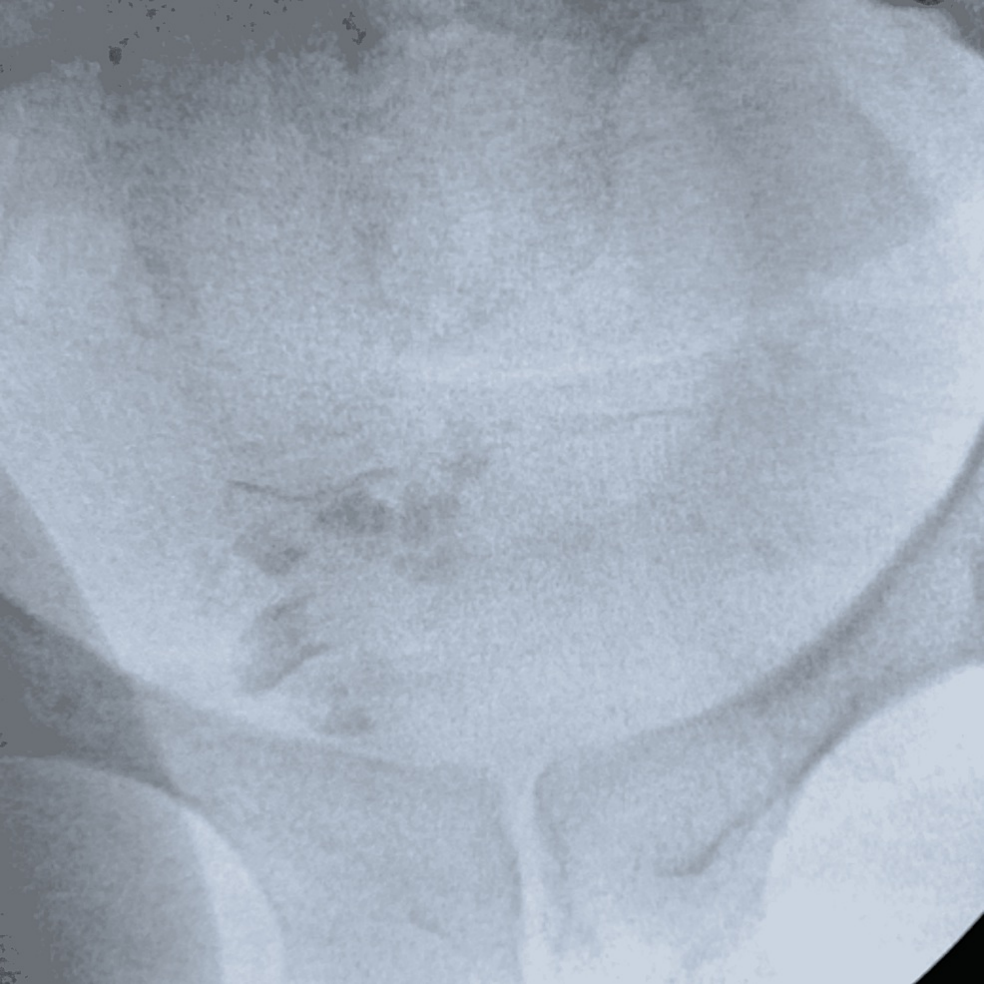
Intraoperative fluoroscopic image shortly after stent removal with residual stone fragments prior to irrigation

**Figure 5:**
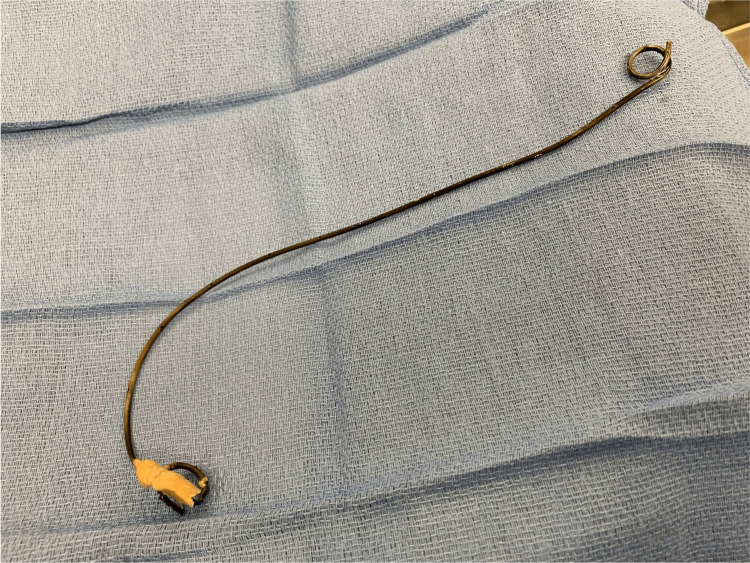
Postoperative image after successful extraction of the calcified ureteral stent

Fluoroscopic imaging and cystoscopy were performed, confirming the pre-operative imaging findings of gross calcifications measuring 3-5 cm on the distal coil of the left-sided stent (Figure 3). A 24-French nephroscope was placed per the urethra. Of note, this scope size can be limited in male patients due to the increased likelihood of urethral stricture formation. The Olympus ShockPulse probe was used to fragment the bulk of the significant stone burden off the distal portion of the stent. This also allowed better visualization of the stent itself. Soltive laser lithotripsy was then performed on the remaining calcifications using a single-use 550-micron fiber (Figure 4). Grasping forceps were then used to remove the stent with fluoroscopy, confirming the uncoiling of the proximal coil and successful, intact stent removal (Figure 5).

The remaining fragments of stone in the bladder were dusted using the Soltive laser, using an open tip catheter for further stabilization, with a pulse energy of 1.5J, frequency of 14Hz, and power of 21W, bringing us to a total laser time of 24.37 minutes. The final fluoroscopic view of the abdomen did not reveal any remaining calcifications in the bladder or overlying the left renal shadow. Cystoscopy confirmed there was no evidence of bladder perforation. Following the procedure, she reported marked improvement in the left flank pain with clear urine output. Upon discharge, her creatinine levels reverted to 1.15 from the initial presentation level of 1.58.

## Discussion

Double-J ureteral stents are one of the most widely used tools in endoscopic urologic practice. They are used following numerous surgical procedures to provide drainage when the ureter is strictured, obstructed, or otherwise dysfunctional [1]. Complications such as stent calcification and de novo stone formation may arise when left in place longer than the recommended period per the stent manufacturer. Although numerous studies have attempted to define the ideal indwelling time, it is not strictly known [2]. Nonetheless, calcification of ureteral stents leads to an increased risk of renal obstruction, urinary infection, and other potentially life-threatening complications that require urgent urologic intervention. 

Endourologic surgery is the preferred modality in managing calcified, retained ureteral stents. Retrieval of severely calcified stents continues to be a challenging task. Mohan-Pillai et al. reported four patients with severely calcified stents and impaired renal function who underwent retrograde ureteroscopy or a combination of percutaneous and ureteroscopic procedures [5]. An average of 2.5 or one to three procedures was carried out for all four participants to be rendered stone-free with subsequent improvement in renal function. Comparably, another technique using a combination of percutaneous nephrolithotomy and ureteroscopy found that patients required an average of 4.2 or two to six procedures performed at one or multiple sessions to treat the severely calcified stents and any accompanying stone burden [6]. 

In recent years, less invasive techniques have been described, including a combined one-step approach, with semirigid ureteroscopy, percutaneous nephrolithotomy, and an open cystolithotomy to manage a completely calcified stent [7]. While other authors may have presented similar methods of endoscopic management, we felt this case demonstrated the feasibility of this technique for those who may have not been taught advanced endourologic management or are practicing outside high-volume academic centers. However, despite current advances, there is a need to integrate novel multimodal procedures, which minimize laser and surgical times, potentially decreasing the risk of iatrogenic complications. Our approach to a distally calcified ureteral stent minimized the laser and surgical time by combining Olympus ShockPulse and Soltive laser lithotripsy. 

## Conclusions

Endourologic surgery is the first choice in managing retained ureteral stents. Successful management requires a combined approach that aims to remove the encrusted stent and any associated stone burden. This report highlights a single-session approach using Olympus ShockPulse and Soltive laser lithotripsy to treat and manage a distally calcified ureteral stent. Initially, fragmenting the bulk of the stone with the Olympus ShockPulse minimizes total laser and surgical times and potentially decreases the risk of iatrogenic complications. 
